# Microarray analysis of human leucocyte subsets: the advantages of positive selection and rapid purification

**DOI:** 10.1186/1471-2164-8-64

**Published:** 2007-03-05

**Authors:** Paul A Lyons, Maria Koukoulaki, Alexander Hatton, Karen Doggett, Hayley B Woffendin, Afzal N Chaudhry, Kenneth GC Smith

**Affiliations:** 1 Department of Medicine, and Cambridge Institute for Medical Research, University of Cambridge School of Clinical Medicine, Addenbrooke's Hospital, Hills Road, Cambridge, CB2 2XY, UK

## Abstract

**Background:**

For expression profiling to have a practical impact in the management of immune-related disease it is essential that it can be applied to peripheral blood cells. Early studies have used total peripheral blood mononuclear cells, and as a consequence the majority of the disease-related signatures identified have simply reflected differences in the relative abundance of individual cell types between patients and controls. To identify cell-specific changes in transcription it would be necessary to profile purified leucocyte subsets.

**Results:**

We have used sequential rounds of positive selection to isolate CD4 and CD8 T cells, CD19 B cells, CD14 monocytes and CD16 neutrophils for microarray analysis from a single blood sample. We compared gene expression in cells isolated in parallel using either positive or negative selection and demonstrate that there are no significant consistent changes due to positive selection, and that the far inferior results obtained by negative selection are largely due to reduced purity. Finally, we demonstrate that storing cells prior to separation leads to profound changes in expression, predominantly in cells of the myeloid lineage.

**Conclusion:**

Leukocyte subsets should be prepared for microarray analysis by rapid positive selection.

## Background

The 'omic' revolution is starting to have a profound impact on the investigation of complex diseases. This technology promises a more rational approach to the treatment of disease as a consequence of the development of new molecular diagnostic tests. These "biomarkers" will impact many areas of the clinical management of disease, including screening patients at risk, classifying patients at presentation, selection of appropriate therapy, monitoring response to treatment, and predicting relapse.

The best example of the application of this technology has been in the field of oncology. Here, microarray-based expression profiling has been widely employed to develop clinically relevant molecular classifiers for many tumour types [1-4]. These classifiers have a better prognostic performance than conventional classifiers based on clinical parameters [5,6], and can predict both the response to therapy [7,8], and the odds of metastasis development [9,10]. The choice of tissue to profile is straightforward in oncology and the whole process is greatly simplified by the fact that tumour material is largely monoclonal. For systemic autoimmune diseases the choice of material to profile is less clear. From a practical perspective the best option is to profile peripheral blood cells, an approach that has been pursued by several groups [11-15].

In these early studies, however, the results have been confounded by the use of unseparated peripheral blood mononuclear cells (PBMC), as many of the expression signatures identified, as well as representing potential changes in cell-type specific gene expression, may simply reflect changes in the cellular constituents of blood. Perhaps the best example of this is the "granulopoiesis" signature identified by Bennett and colleagues, which reflected the fact that patient, but not control, PBMC samples contained significant numbers of immature granulocytes [12]. In the same study the authors also demonstrated an increase in the expression of immunoglobulin genes in some patients [12]. This was also shown to be correlated with the increased number of circulating plasmablasts in these patients (long known to occur in SLE [16]) rather that a change in B cell gene transcription at the cellular level.

To avoid this issue it would be preferable to profile populations of purified cells, but the conditions for performing such cell separations and subsequent analyses are not well established. In particular, it is unclear whether positive or negative selection is best employed for generating purified cells for profiling. It has long been a criticism of positive selection that cross-linking cell surface antigens may well result in cellular activation and altered transcription. This has led to the marketing of negative selection kits yielding "untouched" cells. The evidence supporting the advantages of such negative selection is, however, very sparse [17]. Moreover, no studies have directly addressed the issue of whether specific changes in gene expression actually occur following positive selection. One recent study that used negative selection suggested that it had little effect on gene transcription, though they did not compare their selection protocol to one based on positive selection [18].

Another issue that has not been fully addressed concerns the effect of delay in the cell preparation protocol on gene expression. Two studies have shown that delaying the isolation of PBMC from whole blood for as little as three hours leads to significant systematic changes in gene expression [19,20]. However, it is unclear whether these changes are global in nature or restricted to individual cell types.

To address these issues we have developed and optimised a cell separation protocol utilising sequential rounds of positive selection that enables the isolation for microarray analysis of CD4 and CD8 T cells, CD19 B cells, CD14 monocytes and CD16 neutrophils from a single individual. We compared gene expression in cells isolated in parallel using either positive or negative selection, and demonstrated that with positive selection very few genes change in a systematic way and that the few that do can largely be attributed to contamination, and that negative selection is far inferior due to higher levels of contamination. Finally, we examined the effect on transcription of storing cells prior to separation, and demonstrated that storage leads to profound changes in expression, but only in cells of the myeloid lineage. Thus, leucocyte subsets should be prepared for microarray analysis by rapid positive selection.

## Results

### Purification and microarray analysis of leucocyte subsets

To enable the expression profiling of individual leucocyte subsets we have developed and optimised a cell separation protocol based on sequential rounds of positive selection using magnetic beads. Using this approach sufficient quantities of CD4^+ ^T cells, CD8^+ ^T cells, CD19^+ ^B cells, CD14^+ ^monocytes and CD16^+ ^neutrophils can be purified from a single individual to generate enough RNA for microarray analyses (Table 1). Even those cell populations present at low abundance, such as CD19^+ ^B cells which make up less than 5% of total PBMCs, can be purified to greater than 90% purity (Table 1).

**Figure 1:**
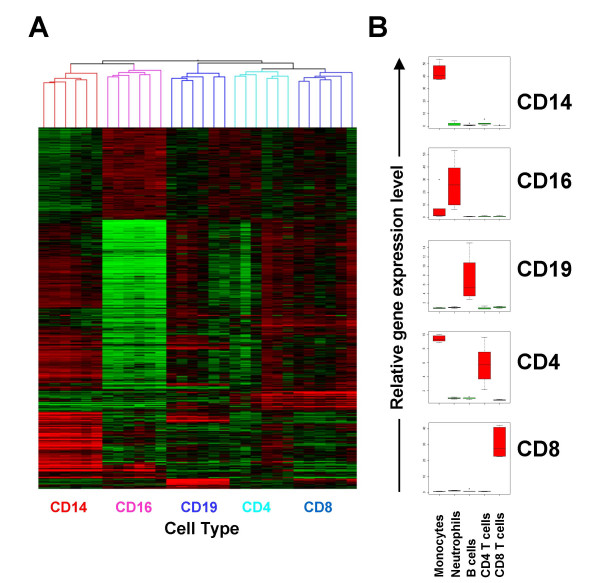
**Microarray analysis of purified cell populations from six normal controls**. (A) Hierarchical clustering of microarray data generated for five cell populations isolated from six normal controls was performed using expression data from 12,022 genes and clusters samples according to cell lineage. (B) Relative expression levels of CD14, CD16, CD19, CD4 and CD8 mRNA in each of the five cell types.

To validate the separation protocol, RNA samples extracted from leucocyte subsets from six normal controls were labelled and hybridised to Affymetrix U133 Plus2 GeneChips (Figure 1). Hierarchical clustering of the samples based on expression data from 12,022 genes determined to be present in all replicates of at least one cell type clusters the samples according to cell lineage (Figure 1A). As an additional validation step we examined the expression profiles of a panel of 39 known cell-specific markers, comprised predominantly of CD antigens [see Additional file 1]. Hierarchical clustering of the samples based on the expression data of these 39 genes again clustered the samples according to cell lineage, with the expression pattern of individual CD antigens being as predicted [see Additional file 2]. For example, mRNA for CD74, the invariant chain, is expressed highly in all CD14^+ ^monocyte and CD19^+ ^B cell samples but not in any other cell type [see Additional file 2].

As a further confirmation of cell purity the expression level of the mRNA encoding each cell surface antigen used for positive selection was measured across all five cell populations (Figure 1B). With the exception of CD4, expression of each of the mRNAs was restricted to the appropriate cell type. In the case of CD4 mRNA, expression was observed not only in CD4^+ ^T cells but also in CD14^+ ^monocytes. This is not unexpected, as monocytes express surface CD4, but clearly suggests that for optimal purities CD4^+ ^T cells should be isolated from a monocyte-depleted sample.

Moreover, cellular expression profiles from any one individual remain stable over time. CD14^+ ^monocyte gene expression profiles measured in three individuals three months apart show a strong correlation (R^2 ^= 0.83 ± 0.06, [see Additional file 3B]) which is of a similar magnitude to that seen between replicates of the same sample, and greater than that seen between samples from different individuals (R^2 ^= 0.83 ± 0.03 and R^2 ^= 0.73 ± 0.06, respectively, [see Additional file 3A and 3C]).

### Positive selection is associated with increased cell purity

**Figure 2:**
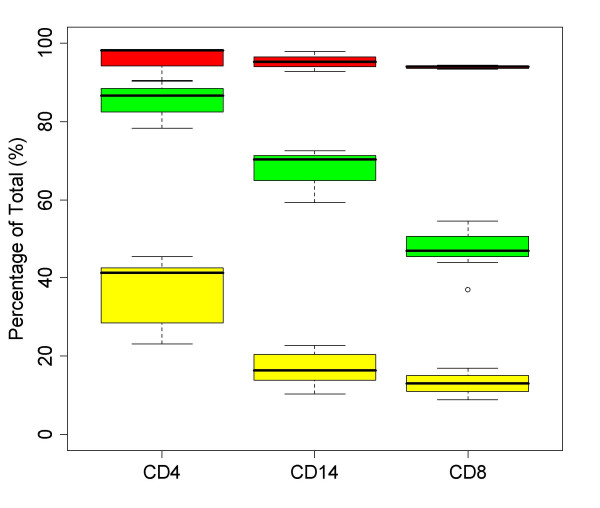
**Positive selection is associated with increased cell purity**. Proportion of cell types (CD4, CD8 and CD14) in PBMC as assessed by flow cytometry prior to separation (yellow boxes). Purity of these cells assessed by flow cytometry following positive (red boxes) or negative (green boxes) selection.

To determine whether purifying cells using positive selection has a significant impact on cellular transcription levels, CD4^+ ^and CD8^+ ^T cells and CD14^+ ^monocytes were purified by either positive or negative selection in parallel. FACS analysis of the purified cell populations showed that in every case positive selection gave better purities than negative selection (CD4: 95.6 ± 4.5 versus 85.1 ± 6.1%, CD14: 95.2 ± 2.5% versus 67.3 ± 7.0%, and CD8: 93.8 ± 0.5% versus 48.5 ± 5.4% for positively and negatively selected cells respectively, Figure 2). In the case of the negatively selected samples, for all three cell types there was a strong correlation between final purity and the relative abundance of all three cell types in total PBMC (R^2 ^= 0.78, Figure 2). The implication of this is that the process of negative selection simply results in an enrichment for, rather than purification of, the cell type being selected.

### The few expression changes associated with selection method can be attributed to contamination with other cell types

**Figure 3:**
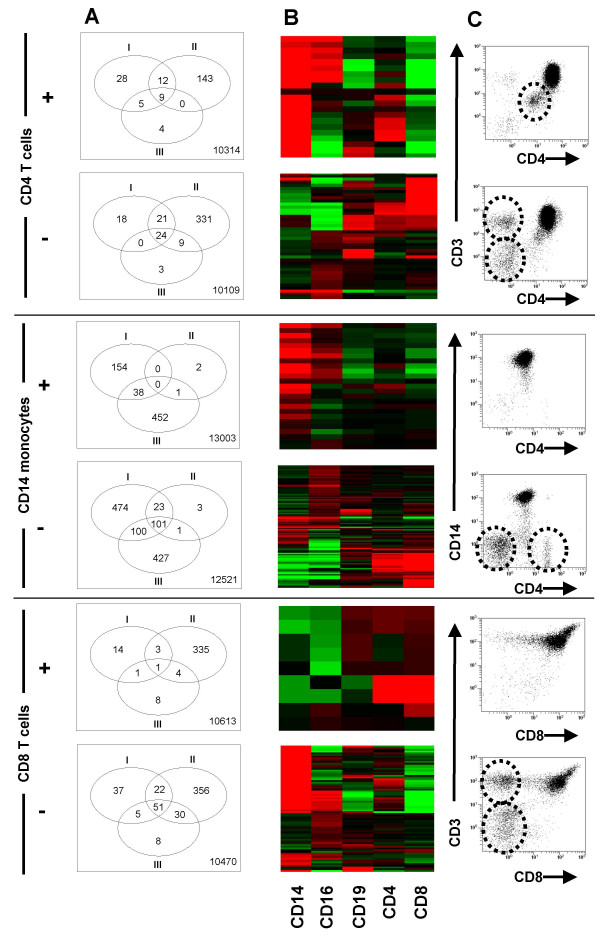
**Differential gene expression following positive or negative selection reflects cellular contamination rather than activation**. (A) Venn diagrams showing number and overlap of statistically significant, differentially expressed genes (as defined in materials and methods) in each independent experiment (roman numerals). The number in the bottom right of each panel is the number of genes whose expression does not change with selection. Genes whose expression changes upon positive (+) or negative (-) selection are shown for each cell type. (B) Heat diagrams show the relative expression pattern of genes significantly changed in 2 out of 3 replicates in 3A on arrays of purified cell subsets from Figure 1A. Red indicates over-expression and green indicates under-expression. (C) Representative flow cytometry profiles for each positively and negatively purified cell type. Putative contaminating cell populations that correlate with the gene expression patterns observed in 3A are ringed (------).

To address the question of whether positive selection alters cellular transcription, RNA from cells isolated in parallel using either positive or negative selection were labelled and hybridised to our custom spotted oligonucleotide microarrays. As shown in Figure 3A the majority of genes called present in all three cell types showed no evidence of statistically significant differential expression. Across the three independent CD4 separations, only 607 out of 10,515 genes (6%) called present were deemed to be differentially expressed between the positively and negatively selected samples. Similar data were obtained for the CD8 (856 of 10,979; 8%) and CD14 (1,776 out of 13,650; 13%) separations. Strikingly, the majority of the differentially expressed genes do not change systematically and are only seen in one out of three experiments (Figure 3A). Relatively few genes were differentially expressed in 2 of 3 independent experiments (CD4: 80 genes (0.7%), CD14: 264 (1.5%) and CD8: 117 (0.9%)), and even fewer in 3 out of 3 experiments (CD4: 33 genes (0.3%), CD14: 101 (0.9%) and CD8: 52 (0.4%)) (Figure 3A).

Of the 80 genes that show consistent differential expression in two out of the three CD4 separations, 26 were over-expressed in the positively selected sample and 54 were over-expressed in the negatively selected sample (Figure 3A). Comparing the expression pattern of the 26 genes over-expressed in the positively selected sample with arrays of leucocyte subsets generated from normal controls (see Figure 1) reveals that they are predominantly expressed in monocytes (Figure 3B). Indeed, FACS analysis of the positively selected CD4^+ ^T cells shows a cell population with the forward and side scatter characteristics of monocytes (ringed in Figure 3C). This suggested that the over-expression of these genes simply represents contamination of the positively selected CD4^+ ^T cells with monocytes rather than transcriptional activation. This problem has subsequently been alleviated by removing the monocytes prior to CD4^+ ^T cell selection which reduces monocyte contamination from 2.9 ± 0.2% to 1.0 ± 0.1% (p = 0.0005).

Similarly for the genes over-expressed in the negatively selected CD4^+ ^T cells, analysis of their leucocyte expression profile on control arrays suggests that this over-expression has again arisen as a consequence of contamination rather than transcriptional activation (Figure 3B). This is further supported by FACS analysis of the negatively selected cells (Figure 3C).

The majority of the differentially expressed genes in CD14 and CD8 separations are over-expressed in the negatively, rather than positively, selected cell populations (positive vs. negative – CD14: 39 vs. 225, CD8: 9 vs. 108, Figure 3A). Strikingly, essentially no genes are increased by positive selection in 3 of 3 experiments (CD14: 0 vs. 101, CD8: 1 vs. 51, Figure 3A). As with CD4 cells, FACS analysis of the negatively selected cell populations together with the leucocyte expression profile of the over-expressed genes supports contamination rather than activation as being the most likely explanation even for this minimal over-expression (Figure 3B and 3C). Overall, the data for the three cell types examined indicates that positive selection does not lead to changes in cellular gene expression patterns, but rather that the limited number of differentially expressed genes seen between positively and negatively selected samples is due to contamination, and this is much more marked in the negatively selected cells.

### Delayed cell purification results in variable but significant transcription changes, particularly in myeloid lineages

A number of studies have demonstrated that storing blood samples prior to isolating whole PBMC has a profound effect on their gene expression profiles [19,21]. It is, however, unclear whether this is a global response in every cell type, or is restricted to specific cell types. To address this question we isolated CD4^+ ^and CD8^+ ^T cells, CD14^+ ^monocytes and CD16^+ ^neutrophils from the same blood sample either immediately post venesection or following storage on ice for four hours. The expression profiles of paired samples where then compared by microarray analysis using custom spotted oligonucleotide arrays.

**Figure 4:**
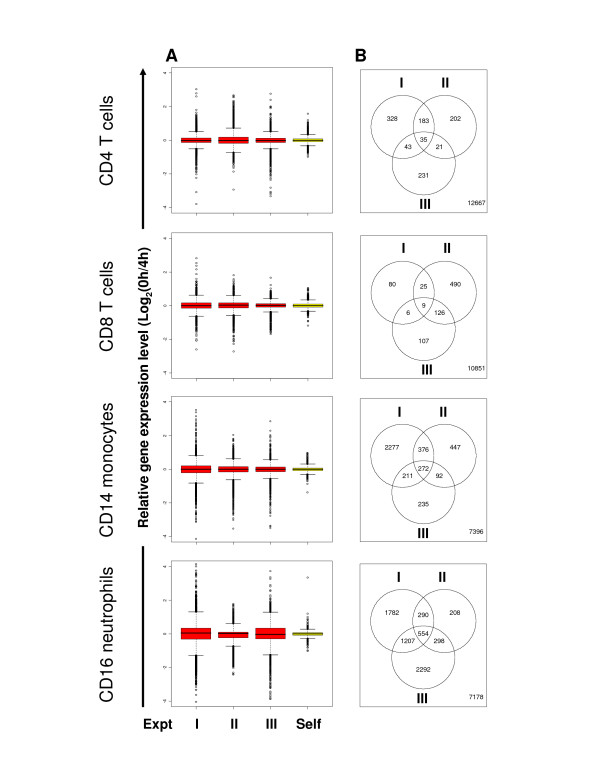
**Delaying separation leads to significant changes in gene expression especially in cells of the myeloid lineage**. Expression profiles were obtained from RNA samples extracted from cells separated immediately following venesection compared to those separated after a four hour delay on ice. Box plots (A) show the change in gene expression between 0 and 4 hours for independent experiments (I – III) and combined self versus self hybridisation data. The Venn diagrams (B) show the number and overlap between genes showing statistically significant differential expression (as defined in the materials and methods).

Storing blood prior to separation has no noticeable effect on the separation process. The final purities for all four cell subsets were not statistically different irrespective of whether the blood was stored prior to separation or not (data not shown). However, storage has a clear effect on RNA levels (Figure 4).

For all four cell types the spread of the log ratio data for freshly isolated versus stored samples was greater than that of the appropriate self versus self hybridisation data (Figure 4A), indicating a significant degree of differential expression between the freshly isolated and stored samples. Analysis of the microarray data shows that for CD4^+ ^T cells 1,043 out of 13,710 (8%), for CD8^+ ^T cells 843 out of 11,694 (7%), for CD14^+ ^monocytes 3,910 out of 11,306 (35%), and for CD16^+ ^neutrophils 6,631 out of 13,809 (48%) genes were statistically differentially expressed between freshly isolated and stored samples in at least one experiment (Figure 4B). The striking difference in number of differentially expressed genes observed in cells of the myeloid lineage compared to cells of the lymphoid lineage was maintained when only genes showing consistent evidence of differential expression (significantly different in two out three independent experiments) were considered. In this case, 282 (2%) and 166 (1%) differentially expressed genes were seen in CD4^+ ^and CD8^+ ^T cells, respectively, compared to 951 (8%) and 2,349 (17%) in CD14^+ ^monocytes and CD16^+ ^neutrophils, respectively (Figure 4B). Based on these observations it is clear that delaying the cell separation process should be avoided if possible, as even a short delay leads to significant alterations in gene transcription. Having said this, delaying separation is much less of an issue if T cells are being studied, especially if the number of samples is high since almost no genes (< 0.25%) were systematically changed by delay in three out of three samples.

## Discussion

For expression profiling to have a practical impact in the management of immune-related diseases, as it is starting to have in oncology, it is essential that it can be applied to peripheral blood cells. Early studies, particularly in SLE, have used total PBMC, and as a consequence the majority of the disease-related signatures identified have simply reflected differences in the relative abundance of individual cell types between patients with disease and controls [12]. To avoid this, and to identify genuine cell specific signatures, is necessary to profile purified leucocyte subsets. However, protocols for doing so are not established.

From a practical perspective the most straightforward approach would be to use sequential rounds of positive selection to isolate individual cell subsets. A critical question that has not been addressed is what effect, if any, the process of positive selection has on cellular transcription profiles. A number of studies have demonstrated that positive selection using magnetic beads appears to have a minimal effect on the activation status of the isolated cells, however none have looked in a systematic manner at gene expression [17,22-26].

To address this we have compared gene expression profiles in CD4^+ ^and CD8^+ ^T cells and CD14^+ ^monocytes isolated by either positive or negative selection. For all three subsets there was no evidence that cross-linking CD4, CD8 or CD14 during the selection process led to widespread changes in gene expression. While on any individual array there was a degree of noise, across all the arrays for each cell type there were very few systematic changes. In each case, the majority of genes that showed a consistent change had a higher level of expression in the negatively selected population. While it is possible that these changes are due to downregulation of the transcription of these genes in the positively selected population, analysis of their expression pattern across a panel of purified leucocyte subsets from a cohort of normal individuals strongly suggests that the elevated expression is due to contamination of the negatively selected population with other cell types. This conclusion is supported by FACS analysis of the negatively selected cell populations which show the presence of contaminating cell types consistent with the expression data.

Contamination of the positively selected cells was only an issue for one cell type, namely the CD4^+ ^T cells, which consistently contained low levels of CD14^+ ^monocytes. This is simply a consequence of the expression of CD4 by monocytes, and can be efficiently eliminated by carrying out the CD4 selection on CD14-depleted PBMC. In a recent study Du and colleagues used negative selection to isolate individual cell subsets from peripheral blood, and identified 269 probe sets that were significantly differentially expressed between the cell subsets in their study [18]. An identical analysis of our own data (parametric analysis of variance, p < 0.05 with Bonferroni correction followed by a Student-Newman-Keuls post-hoc test) identified 2,641 probe sets [see Additional file 4] that are differentially expressed between CD4^+ ^and CD8^+ ^T cells, CD19^+ ^B cells, CD14^+ ^monocytes and CD16^+ ^neutrophils. The 269 probe sets identified by Du et al represent 195 unique genes of which 141 would differentiate the cell types analysed in our study (as we did not isolate natural killer cells or platelets). Of these 141 genes, 94 are also identified by our analysis, and a further 36 show the correct differential expression pattern although it fails to reach statistical significance using the conservative Bonferroni correction for multiple testing. The high degree of similarity between the two studies, despite the different purification strategies, further support the suggestion that positive selection does not lead to significant alterations in gene expression. On the basis of these observations it is clear that positive selection is preferable to negative selection for generating cells for microarray analysis as it yields higher cell purities and negligible changes in gene expression.

Another issue that could potentially confound microarray analysis is variation introduced during the blood handling process, and in particular as a consequence of delays prior to cell separation. Two studies have shown that even short delays between blood being taken and the separation process starting leads to significant systematic variation in gene expression in unseparated PBMC [19,20]. In this study we have extended these observations to look at the effect of delaying cell separation on gene expression in individual purified cell types. It is clear from the data that even a short delay in processing the blood sample results in significant changes in gene expression in cells of the myeloid lineage. However, this is not a practical problem when looking at cells of the T cell lineage, especially where large sample numbers are available, as the number of genes showing a systematic change in expression is negligible.

## Conclusion

On the basis of these findings the use of positive selection has no adverse influence on cellular transcription, at least for the antibody-receptor combinations examined. Thus, for microarray analysis of purified cell subsets where high purity is essential it may be preferable to use positive rather than negative selection. In addition, delay in the selection process should be minimised and standardized, especially if myeloid cells are to be studied.

## Methods

### Cell Separations

Blood samples (100 ml) were collected into 4% sodium citrate. Within 15 min of collection the blood was diluted 1:2 with MACS rinsing buffer (1× phosphate buffered saline (PBS), 2 mM EDTA) and centrifuged on Histopaque 1077 (Sigma) at 900 *g *for 20 min at room temperature. Following centrifugation, the PBMC at the interface were removed, washed twice with MACS rinsing buffer, and then resuspended in 50 ml MACS running buffer (1× PBS, 2 mM EDTA, 0.5% BSA).

#### Positive selection

The PBMC sample was split into two aliquots and CD14 monocytes were isolated from one aliquot and CD19 B cells from the other by magnetic cell sorting using CD14 and CD19 microbeads (Miltenyi Biotec) according to the manufacturer's instructions. CD4 and CD8 T cells were then isolated from the CD14 and CD19 negative fractions, respectively, by magnetic cell sorting using CD4 and CD8 microbeads as described by the manufacturer. The positive selection protocol as outlined takes less than 5 hours from time of blood collection to RNA extraction.

CD16 neutrophils were obtained as follows. Following centrifugation on Histopaque 1077 the red cell/granulocyte pellet was incubated with red cell lysis buffer (155 mM NH_4_Cl, 12 mM NaHCO_3_, 0.1 mM EDTA) on ice for 30 min. Following red cell lysis the granulocytes were recovered by centrifugation, washed with MACS rinsing buffer, and then resuspended in 50 ml MACS running buffer. Neutrophils were then isolated by magnetic cell sorting using CD16 microbeads as described by the manufacturer.

#### Negative selection

"Untouched" CD4 and CD8 T cells and CD14 monocytes were purified from PBMC by negative selection using CD4 T cell, CD8 T cell and monocyte isolation kits (Miltenyi Biotec) according to the manufacturer's instructions. Briefly, PBMC were resuspended in MACS running buffer at 2 × 10^8 ^cells/ml and labelled with the appropriate negative selection biotin-antibody cocktail for 10 min at 4–8°C. Labelled cells were then diluted to 1 × 10^8 ^cells/ml in MACS running buffer and incubated with anti-biotin microbeads for an additional 15 min at 4–8°C. The cells were then washed and resuspended in 500 μl MACS running buffer prior to magnetic cell sorting using an autoMACS (Miltenyi Biotech).

### Antibodies and FACS analysis

Cell purities pre- and post-separation were measured by flow cytometry using a FACSCalibur (BD Biosciences). FITC-labelled anti-CD4, anti-CD15 and anti-CD20, PE-labelled anti-CD3, anti-CD14, anti-CD19 and anti-CD16, and APC-labelled anti-CD8 monoclonal antibodies were purchased from BD Biosciences.

### RNA extraction, labelling and microarray hybridisation

RNA was extracted using RNEasy mini kits (Qiagen) according to the manufacturer's instructions. RNA quality was assessed using an Agilent Bioanalyser 2100 and quantified by spectrophotometry using a NanoDrop ND-1000 spectrophotometer.

Custom microarrays were printed at the Centre for Microarray Resources, Department of Pathology, University of Cambridge using 50 mer olignucleotide probes representing 25,342 genes and control probes [27]. For microarray hybridisations, 250 ng of total RNA was converted into double stranded cDNA and then 100 ng of cDNA was random prime labelled with either Cy3-dCTP or Cy5-dCTP as described [28]. Appropriate Cy3- and Cy5-labelled targets were pooled, precipitated and resuspended in 250 μl hybridisation buffer (40% formamide, 5× SSC, 5× Denhardt's solution, 1 mM sodium pyrophosphate, 50 mM Tris pH 7.4, 0.1% SDS). Pooled targets were denatured at 95°C for 5 min, incubated at 50°C for 5 min, centrifuged at 13,000 rpm for 5 min, and then hybridised to custom spotted oligonucleotide microarrays at 42°C for 16 h on a Lucidea SlidePro hybridisation station (GE Healthcare). Following hybridisation arrays were washed at room temperature in 1× SSC/0.2% SDS for 5 min, 1× SSC for 5 min, and 0.1× SSC for 5 min, and then dried by centrifugation at 500 *g *for 2 min. Arrays were scanned at 10 micron resolution using an Agilent G2565B scanner.

### Microarray data analysis

Raw image data was extracted using Koadarray v2.4 software (Koada Technology), probes were called present if they had a spot confidence value > 0.3 in at least one channel. Background subtracted intensity values for all probes considered present were imported into R where within-print-tip Lowess normalisation and the identification of statistically significant, differential gene expression was performed using the LIMMA library in the Bioconductor software package [29]. To correct for multiple testing p-values were adjusted using the method of Benjamini and Hochberg by setting the false discovery rate to 10% [30].

### Affymetrix analysis

For hybridisation to Affymetrix GeneChips 100 ng of total RNA was converted into double-stranded cDNA using the SMART cDNA synthesis kit (Clontech) except that a T7 tag was added to the primer used for first strand cDNA synthesis. Biotin-labelled cRNA was generated, fragmented, and hybridised onto Affymetrix Human Genome U133 Plus 2 arrays according to the manufacturer's instructions. Following hybridisation and washing, the arrays were scanned using a GeneChip Scanner 3000. Raw data files were imported into GeneSpring v7.2 and RMA normalized [31] prior to further analysis. Genes were called present if they had a signal intensity greater than 150 fluorescence units following normalization. Hierarchical clustering of the RMA normalized Affymetrix data was carried out in GeneSpring using the Pearson correlation as the measure of similarity.

## Abbreviations

SLE: Systemic lupus erythematosus

PBMC: Peripheral blood mononuclear cells

FACS: Fluorescence-activated cell sorting

## Authors' contributions

PAL conceived and co-ordinated the study, performed the data analysis and drafted the manuscript. MK, KD, AH and HBW carried out the laboratory component of the study. ANC contributed to the data analysis. KGCS participated in the design of the study, contributed to the data analysis, and helped to draft the manuscript. All authors read and approved the final manuscript.

## Supplementary Material

Additional file 1 Cell type specific gene list. This gene list was used as the basis for the hierarchical clustering shown in Additional file 2. Click here for file

Additional file 2 Hierarchical clustering of purified cell samples on the basis of CD antigen expression groups samples by cell lineage. Hierarchical clustering using expression data from 39 genes was performed using the Pearson correlation as the measure of similarity. Click here for file

Additional file 3 RNA samples extracted from the same individual over time show stable expression profiles. (A) Representative plot showing the correlation between array data from independent labellings of the same CD14 monocyte RNA sample. (B) Representative plot showing the correlation between array data from CD14 monocyte RNA samples extracted from the same individual 3 months apart. (C) Representative plot showing the correlation between array data for CD14 monocyte RNA samples extracted from different individuals. In each case mean ± SD R^2 ^values are shown. All samples were hybridised to spotted oligonucleotide microarrays comprised of probes representing 25,342 known genes or control elements. Click here for file

Additional file 4 Genes showing cell type specific expression patterns. Statistical analysis of the microarray dataset shown in Figure 1 (parametric analysis of variance, p < 0.05 with Bonferroni correction followed by a Student-Newman-Keuls post-hoc test) identified 2,641 probe sets that are differentially expressed between CD4^+ ^and CD8^+ ^T cells, CD19^+ ^B cells, CD14^+ ^monocytes and CD16^+ ^neutrophils. Click here for file

## Figures and Tables

**Table 1:** Results of cell separations and RNA extractions performed on blood samples from normal controls (n = 6).

	Yield (Cell Number × 10^6^/50 ml)	Purity (%)	RNA Yield (μg/50 ml blood)
CD4 T cells	9.9 ± 1.9	96.8 ± 2.7	3.61 ± 1.8
CD8 T cells	6.0 ± 1.7	87.2 ± 1.8	3.01 ± 0.7
CD14 Monocytes	6.6 ± 2.8	97.6 ± 0.7	3.43 ± 1.8
CD16 Neutrophils	126.0 ± 24.8	99.1 ± 0.1	4.37 ± 2.9
CD19 B cells	2.1 ± 1.2	91.1 ± 2.9	1.64 ± 0.6
