# Supplementary material for: Microarray analysis of human leucocyte subsets: the advantages of positive selection and rapid purification
Source: BMC Genomics. 2007 Mar 5;8:64. doi: 10.1186/1471-2164-8-64 (PMC1828063; doi:10.1186/1471-2164-8-64)
Supplement: Additional file 2 — Hierarchical clustering of purified cell samples on the basis of CD antigen expression groups samples by cell lineage. Hierarchical clustering using expression data from 39 genes was performed using the Pearson correlation as the measure of similarity. [file 1471-2164-8-64-S2.ppt]

## Slide 1
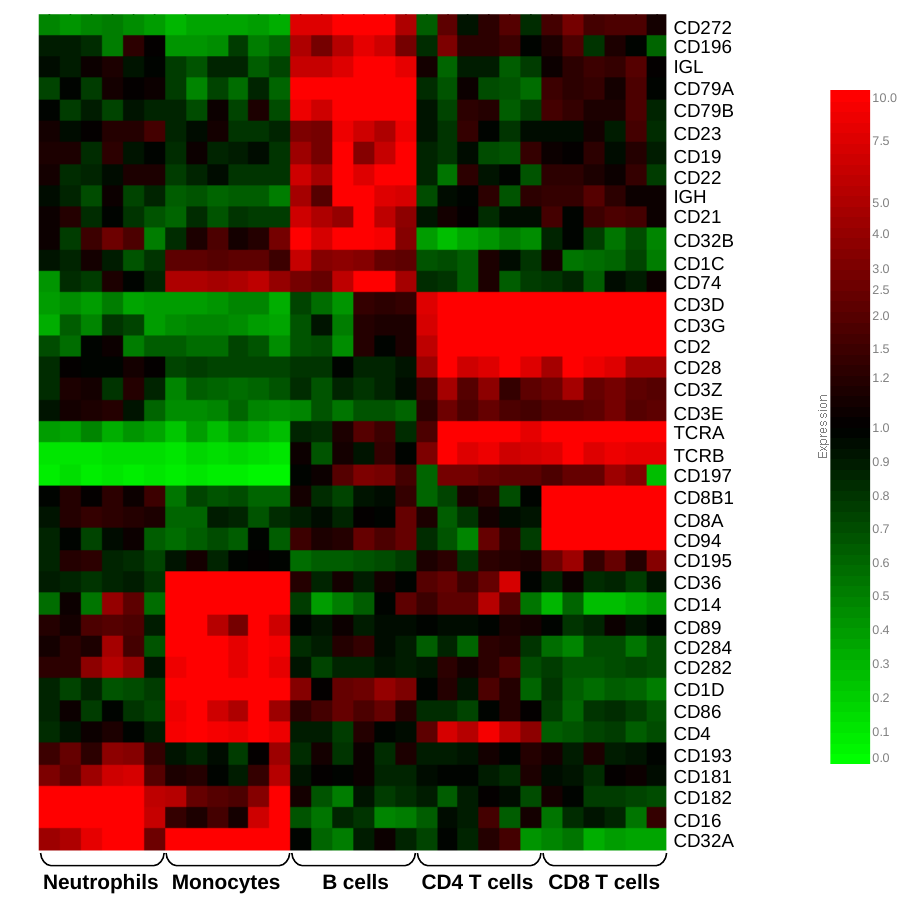

CD272
CD196
IGL
CD79A
CD79B
CD23
CD19
CD22
IGH
CD21
CD32B
CD1C
CD74
CD3D
CD3G
CD2
CD28
CD3Z
CD3E
TCRA
TCRB
CD197
CD8B1
CD8A
CD94
CD195
CD36
CD14
CD89
CD284
CD282
CD1D
CD86
CD4
CD193
CD181
CD182
CD16
CD32A
Neutrophils
Monocytes
B cells
CD4 T cells
CD8 T cells
