# Supplementary figures and images for: Microarray analysis of human leucocyte subsets: the advantages of positive selection and rapid purification
Source: BMC Genomics. 2007 Mar 5;8:64. doi: 10.1186/1471-2164-8-64 (PMC1828063; doi:10.1186/1471-2164-8-64)

## Slide 1
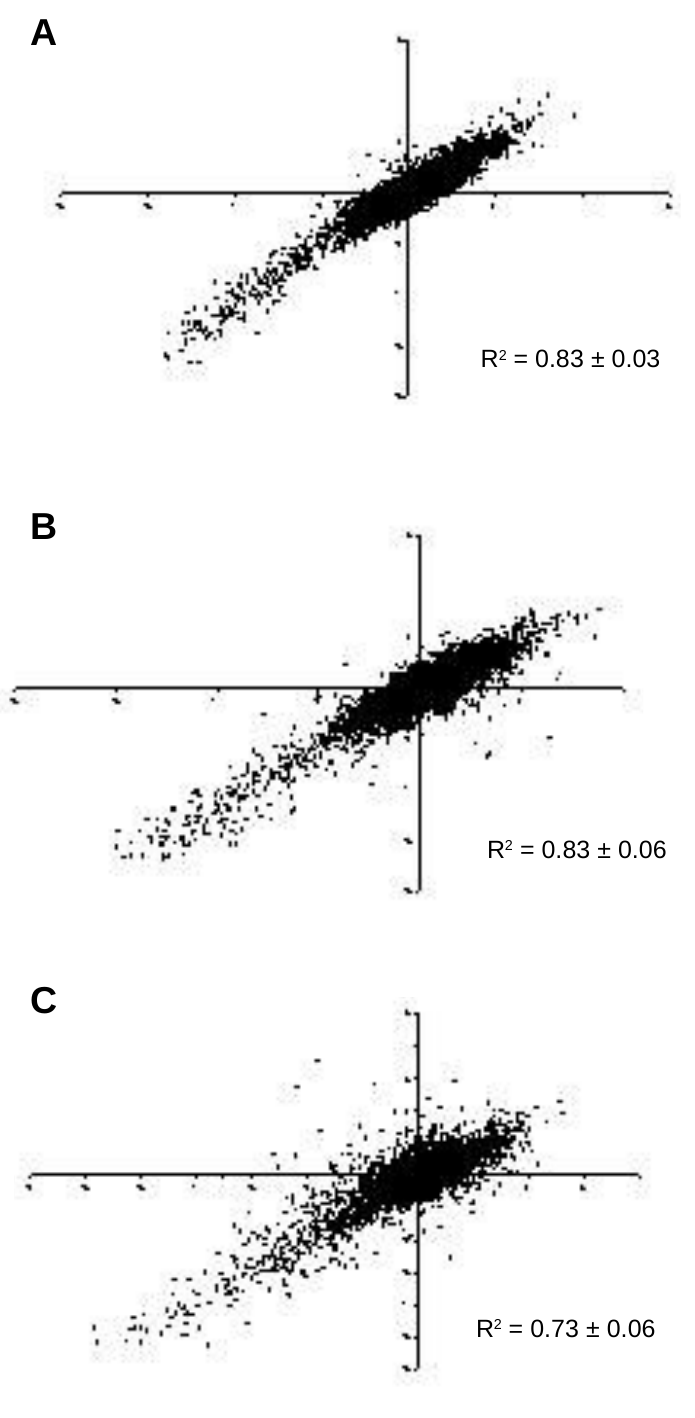

A
R2 = 0.83 ± 0.03
B
R2 = 0.83 ± 0.06
C
R2 = 0.73 ± 0.06

Supplement: Additional file 3 — RNA samples extracted from the same individual over time show stable expression profiles. (A) Representative plot showing the correlation between array data from independent labellings of the same CD14 monocyte RNA sample. (B) Representative plot showing the correlation between array data from CD14 monocyte RNA samples extracted from the same individual 3 months apart. (C) Representative plot showing the correlation between array data for CD14 monocyte RNA samples extracted from different individuals. In each case mean ± SD R2 values are shown. All samples were hybridised to spotted oligonucleotide microarrays comprised of probes representing 25,342 known genes or control elements. [file 1471-2164-8-64-S3.ppt]
